# Evolution of the intercontinental disjunctions in six continents in the *Ampelopsis *clade of the grape family (Vitaceae)

**DOI:** 10.1186/1471-2148-12-17

**Published:** 2012-02-08

**Authors:** Ze-Long Nie, Hang Sun, Steven R Manchester, Ying Meng, Quentin Luke, Jun Wen

**Affiliations:** 1 Key Laboratory of Biodiversity and Biogeography, Kunming Institute of Botany, Chinese Academy of Sciences, Kunming, Yunnan 650204, China; 2 Florida Museum of Natural History, University of Florida, Gainesville, FL 32611, USA; 3 Institute of Tibetan Plateau Research at Kunming, Kunming Institute of Botany, Chinese Academy of Sciences, Kunming, Yunnan 650204, China; 4 East African Herbarium, National Museums of Kenya, Nairobi 00502, Kenya; 5 Department of Botany, National Museum of Natural History, MRC 166, Smithsonian Institution, Washington, DC 20013-7012, USA

## Abstract

**Background:**

The *Ampelopsis *clade (*Ampelopsis *and its close allies) of the grape family Vitaceae contains ca. 43 species disjunctly distributed in Asia, Europe, North America, South America, Africa, and Australia, and is a rare example to study both the Northern and the Southern Hemisphere intercontinental disjunctions. We reconstruct the temporal and spatial diversification of the *Ampelopsis *clade to explore the evolutionary processes that have resulted in their intercontinental disjunctions in six continents.

**Results:**

The Bayesian molecular clock dating and the likelihood ancestral area analyses suggest that the *Ampelopsis *clade most likely originated in North America with its crown group dated at 41.2 Ma (95% HPD 23.4 - 61.0 Ma) in the middle Eocene. Two independent Laurasian migrations into Eurasia are inferred to have occurred in the early Miocene via the North Atlantic land bridges. The ancestor of the Southern Hemisphere lineage migrated from North America to South America in the early Oligocene. The Gondwanan-like pattern of intercontinental disjunction is best explained by two long-distance dispersals: once from South America to Africa estimated at 30.5 Ma (95% HPD 16.9 - 45.9 Ma), and the other from South America to Australia dated to 19.2 Ma (95% HPD 6.7 - 22.3 Ma).

**Conclusions:**

The global disjunctions in the *Ampelopsis *clade are best explained by a diversification model of North American origin, two Laurasian migrations, one migration into South America, and two post-Gondwanan long-distance dispersals. These findings highlight the importance of both vicariance and long distance dispersal in shaping intercontinental disjunctions of flowering plants.

## Background

Understanding the underlying mechanisms for the evolution of wide-ranging disjunct patterns has long been a major focus of biogeography [1-5]. Taxa disjunct at the global level involving both Northern and Southern Hemisphere are particularly informative because their histories may have general implications for other groups. Biogeographic history in the Northern Hemisphere is complicated, but has usually been explained by the widespread distribution of the Boreotropical flora in the Eocene and followed by appearance of more temperate forest elements during the mid-Tertiary extirpations of thermophilic elements in response to climatic cooling episodes of the late Eocene and the Plio-Pleistocene [6-11]. The Southern Hemisphere is often interpreted to show a vicariance pattern attributed to the sequential breakup of Gondwanan landmasses [12]. Recent studies on *Nothofagus *have demonstrated the relevance of long distance dispersal rather than vicariance in shaping Gondwanan distributional patterns [13-16].

**Figure 1:**
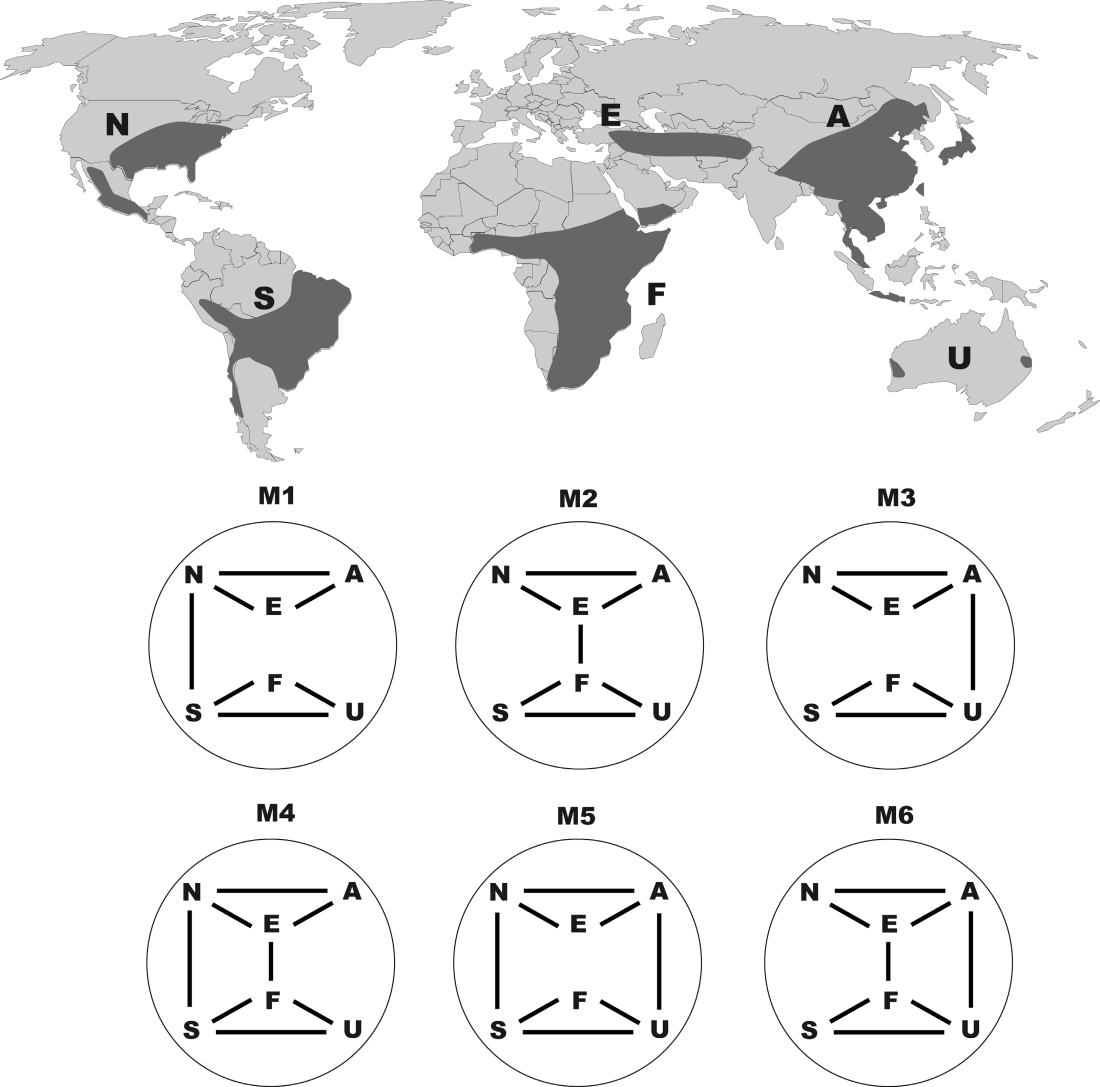
**Delimitation of the six areas for taxa in the *Ampelopsis *clade and the schematic models used in the Lagrange analyses**. N = North America; A = eastern Asia; E = Europe to central Asia; F = Africa; S = South America; and U = Australia.

Commonly three main routes for the migration of taxa between the Northern and the Southern Hemisphere have been recognized (Figure 1). The first is the opening of biotic exchanges between North and South America at various times in the Tertiary [1,17]. The second hypothetical migration route is the trans-Tethyan dispersal between Europe and Africa [18]. The third is less common concerning the possible route between Asia and Australia in the Miocene and later [18,19]. These three routes can be viewed as alternative hypotheses for the ex situ origin of elements of global diversity.

There are many examples of global distribution between the Southern and the Northern Hemisphere, particularly in pantropical families (e.g., Rubiaceae, Annonaceae, Lauraceae) or in many large cosmopolitan genera, such as *Ranunculus *[20], *Senecio *[21], and *Lobelia *[22,23]. Nevertheless, all of them have continuous distribution from the tropics to the temperate zones. Molecular studies usually show a very complex disjunct history of large taxa with relatively low resolution [24,25]. There are also some taxa with relatively few species that exhibit an intercontinental disjunction involving both the Northern and the Southern Hemisphere. However, the disjunction in such taxa usually involves only one or two northern or southern landmasses. Examples include *Caltha *(Ranunculaceae) with a global disjunction but absent from Africa [26] and *Thamnosma *(Rutaceae) disjunct between North America and Africa [27]. Of great interest, we have recently found a truly global disjunct pattern in a small group including *Ampelopsis *Michx. and its relative taxa from the grape family, Vitaceae [28-30].

Vitaceae is a well-known group of flowering plants having a largely pantropical distribution in Asia, Africa, Australia, the neotropics, and the Pacific islands, with only a few genera in temperate regions [31-33]. As currently circumscribed, *Ampelopsis *is one of the few genera mostly restricted to the north temperate zone. It has approximately 25 species disjunctly distributed in Eurasia (c. 22 spp.) and North and Central America (3 spp.). Recent phylogenetic analyses based on plastid or nuclear sequences revealed that there are at least two disjunctions between the New and the Old World in the genus [28,29]. More interestingly, both chloroplast and nuclear data clearly suggested that the African *Rhoicissus *Planch. and the South American *Cissus striata *Ruiz & Pav. complex form a clade nested within the paraphyletic *Ampelopsis *[28,29]. *Rhoicissus *consists of about 12 species endemic to tropical and southern Africa. The *Cissus striata *complex contains four species from South America [29,34]. Furthermore, the Australian genus *Clematicissus *Planch. seemed to be closely related to the *Cissus striata *- *Rhoicissus *clade based on plastid data [30,35]. There are only two *Clematicissus *species known from Australia: *C. opaca *from Australia's eastern region and *C. angustissima *from the west coast [30]. Therefore, the Northern Hemisphere *Ampelopsis *and its three close relatives (*Rhoicissus*, *Clematicissus *and the *Cissus striata *complex) from the Southern Hemisphere (hereafter referred to as the *Ampelopsis *clade) demonstrate an unusual global intercontinental disjunct pattern involving six continents (Figure 1). Yet morphologically, the *Ampelopsis *clade seems to be heterogeneous, such as in having 4-7-merous flowers and fleshy to dry fruits [29].

The *Ampelopsis *clade offers a good opportunity to explore the origin and evolution of the global intercontinental disjunct pattern in flowering plants, especially concerning both Northern and Southern Hemisphere intercontinental disjunctions. A hierarchical global distribution was predicted by our previous studies, but with limited sampling [28,29]. We herein employ phylogenetic, molecular dating, and biogeographic methods to reconstruct the evolutionary history of the *Ampelopsis *clade based on a comprehensive sampling scheme using four plastid regions (*trnL-F*, *rps16*, *psbA-trnH*, and *atpB-rbcL*).

## Methods

### Taxon sampling, DNA sequencing, and phylogenetic analyses

We sample 28 of the 43 species (65%) of the *Ampelopsis *clade including all three North American *Ampelopsis *species, 15 of the 22 Eurasian *Ampelopsis*, five of the 12 *Rhoicissus *species from Africa, three of the four species from the *Cissus striata *complex from South America, and the two Australian *Clematicissus *species (Additional file 1, Table S1). The sampling covers the entire extant geographic range of *Ampelopsis *and its close relatives from both the Northern and Southern Hemisphere. But we still have some missing taxa in our sampling scheme, such as *Rhoicissus *with only 41% species sampled. However, each group is supposed to be monophyletic based on morphological, biogeographic, and molecular evidence [28-30,34,36]. The missing taxa in our sampling should have little effect in the present study that focused on phylogenetic relationships among genera and biogeographic evolution at the intercontinental level. In order to place our analyses of the *Ampelopsis *clade in a broad framework on the family level, we sampled 66 additional taxa from the other major groups in Vitaceae (i.e., the *Vitis *- *Parthenocissus *- *Ampelocissus *clade, the core *Cissus *clade, and the *Cyphostemma *- *Caryatia *- *Tetrastigma *clade) plus three *Leea *species of Leeaceae based on previous investigations [28,29].

Total DNAs were extracted from silica gel dried leaves using the Dneasy Plant Mini Kit (QIAGEN, Crawley, UK). Amplification and sequencing followed Soejima and Wen (2006) for *trnL-F*, *rps16*, and *atpB-rbcL*, and Meng *et al*. [37] for *psbA-trnH*. DNA sequences were assembled using Sequencher v4.1.4 (Gene Codes Corp., Ann Arbor, Michigan, USA). Sequence alignment was initially performed using MUSCLE 3.8.31 [38] in the multiple alignment routine followed by manual adjustment in Se-Al v2.0a11 (http://tree.bio.ed.ac.uk/software/seal/). The chloroplast genome is generally considered as one unit without recombination although there have been reports of recombination in the chloroplast genome [39]. Therefore, we combined all the plastid data (*trnL-F*, *rps16*, *psbA-trnH*, and *atpB-rbcL*) in our analysis. The combined plastid data were analyzed using Bayesian inference as implemented in MrBayes 3.1.2 [40]. The best-fit model of nucleotide substitution (GTR + I + G) was determined by MrModelTest 2.3 [41] using the Akaike Information Criterion (AIC). Variation of gaps in our sequences is not complicate. A total of 31 binary characters were coded for gaps according to Simmons and Ochoterena [42] and separated into independent partition in all analyses. Bayesian tree topology and posterior probabilities (PP) were determined from two independent runs of four incrementally heated chains. Runs were performed for 5 million generations with sampling of trees every 500^th ^generation. When the log-likelihood scores were found to have stabilized, a consensus tree was calculated after omitting the first 10% of trees as burn-in.

### Divergence time estimation

For molecular dating analyses, the strict molecular clock model was rejected from our dataset based on a likelihood ratio test performed in PAUP* [43]. Therefore, we estimated node ages within the *Ampelopsis *group using a Bayesian relaxed clock model as implemented in BEAST v1.6.1 [44]. We largely followed the dating strategies in Nie *et al*. (2010), which analyzed diversification in *Parthenocissus *of Vitaceae. After optimal operator adjustment as suggested by the output diagnostics from several preliminary BEAST runs, two final independent runs (each 50 million generations) were performed on a cluster of Mac XServes used for analysis of biological data at the Smithsonian Institution (http://topazweb.si.edu). Tracer version 1.5 was used to check for convergence between the runs [44]. After discarding the first 10% samples as burn-in, the trees and parameter estimates from the two runs were combined using LogCombiner 1.6.1 [44]. Results were considered reliable once the effective sampling size (ESS) for all parameters exceeded 200 as suggested by the program manual [45]. The samples from the posterior were summarized on the maximum clade credibility tree using the program TreeAnnotator 1.6.1 [44] with posterior probability limit of 0.5 and mean node heights summarized.

Fossil seeds of Vitaceae can be differentiated to the generic level [46,47]. The oldest best preserved seed fossil of the family is from the late Paleocene of the Beicegel Creek locality in North Dakota. This fossil is undoubtedly assigned to *Ampelocissus *s.l. (as *A. parvisemina *Chen & Manchester) and is easily distinguished from all other vitaceous genera by its long, parallel ventral infolds and a centrally positioned oval chalazal scar [46]. Since the *Ampelocissus *s.l. is not monophyletic with *Vitis *nested within it [28], the *A. parvisemina *fossil thus may represent an early member of the *Ampelocissus *clade retaining some characters shared with its common ancestor to *Vitis *[46]. The stem age of the *Ampelocissus - Vitis *clade was thus fixed at 58.5 ± 5.0 million years ago (Ma).

For the root age of Vitaceae, Nie *et al*. (2010) fixed the split between Vitaceae and *Leea *as 85 ± 4 based on the estimated age of 78-92 Ma by Wikström *et al*. (2001). Recently, Bell *et al*. (2010) reported an estimate ranging from 65 (45 *- *81) to 48 (21 *- *79) Ma for the crown age *Vitis *- *Leea *clade, which is roughly consistent with the earliest fossil evidences of Vitaceae in the Palaeocene [46]. However, their results may have underestimated for Vitaceae because the oldest fossil of *A. parvisemina *is undoubtedly assigned to the *Ampelocissus *s.l. within Vitaceae and the family is predicted to have a Cretaceous history in view of its basal position in the rosids and the presence of Cretaceous rosid fossils [48]. The time estimates of angiosperms by Magallón and Castillo (2009) also suggested a pre-Tertiary origin as 90.65 (90.47 *- *90.84) to 90.82 (90.64 *- *91) Ma for Vitaceae. The inferences from Magallón and Castillo (2009) and Wikström et al. (2001) are close, although the later was criticized for the nonparametric rate smoothing method and for calibrating the tree using only a single calibration point. Therefore, we used the estimate from Magallón and Castillo (2009) and set the normal prior distribution of 90.7 ± 1.0 Ma for the stem age of the family. A low standard error was used because of the narrow 95% confidence from Magallón and Castillo (2009).

### Ancestral area reconstruction

Several methods have been recently proposed that take into account of genetic branch lengths, phylogenetic uncertainty, and branch length uncertainty for reconstructing distributional change through evolutionary time, using either maximum likelihood [49] or Bayesian inference [50]. The ancestral area of the *Ampelopsis *clade was reconstructed with the likelihood analysis using the program Lagrange version 20110117 [49,51]. Unlike the DIVA method [52], this likelihood approach incorporates an explicit dispersal-extinction-cladogenesis (DEC) model of dispersal routes available at historical intervals correlating stochastic events with lineage persistence [49]. The likelihood analysis is prone to estimate wide ancestral ranges for early-branching lineages [53-55]. In our case, ancestral ranges were assumed to include no more than two areas since all extant species in the *Ampelopsis *clade are restricted into only one area. Moreover, spatial and temporal constraints (e.g., area distances, continent connections, dates of geological origin) may be imposed in the DEC model estimation, providing a more accurate estimation of the ancestral ranges and hypothesis testing of different geographic scenarios. We did not conduct the Bayesian calculation of ancestral geographic distributions with standard continuous-time Markov chains (CTMCs), because geologic information (e.g., the presence and dissolution of land bridges and island chains) is not explicitly incorporated into the analyses.

We herein used the likelihood method to test a null model and six alternative biogeographic scenarios (Figure 1) based on the hypothesized dispersal or migration routes between the Northern and the Southern Hemisphere. Six areas were delimited by continental divisions and the extant distributions of the *Ampelopsis *clade: 1) N *- *North America including Central America; 2) S *- *South America; 3) F *- *Africa; 4) A *- *eastern Asia; 5) E *- *Europe to central Asia; and 6) U *- *Australia (Figure 1). The unconstrained null model (M0) assumes that spatial and temporal distribution has no effect on biogeographic patterns of evolution and allows geographic ranges to include any possible combination of continents and permits direct dispersal between any area pairs. The M1 model favors a migration route from North to South America (N *- *S) with the biogeographic connections between Europe and Africa (E *- *F) and eastern Asia and Australia (A *- *U) excluded from our analyses. Similarly, the M2 model considers the connection between E *- *F and did not allow other possibilities. The migration route between Asia and Australia (M3) seems less likely, but we still considered it in our analyses as a comparison. We also test models that allow two connections between the Northern and Southern hemispheres (M4 - M6 in Figure 1). Following Ree *et al*. (2005), the results between models were assessed by directly comparing their log-likelihood scores. The conventional cut-off value over two log-likelihood units was considered statistically significant, and models with lower likelihood score were rejected [56,57].

## Results

**Figure 2:**
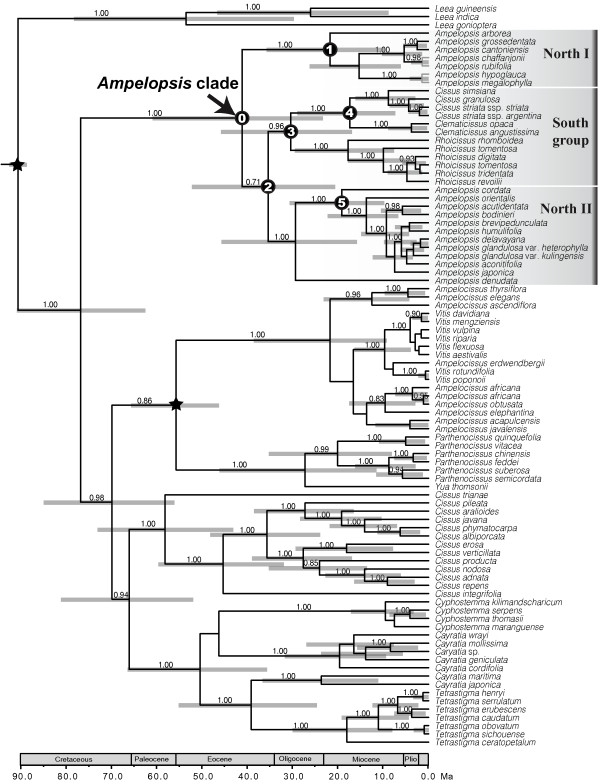
**Maximum clade credibility tree inferred with BEAST, with the 95% highest posterior density indicated by gray bars**. Nodes of interests were marked as 0 to 5 as in Table 1; and calibrations are indicated with black stars. Values above branches represent Bayesian posterior probabilities.

The total length of the aligned data matrix is 3933 bp. The Bayesian consensus tree is highly congruent with the maximum clade credibility tree obtained from BEAST and the later is shown in Figure 2 with PP support values > 0.50. Our results support the monophyly of the *Ampelopsis *clade with three major lineages resolved within the *Ampelopsis *clade (Figure 2). Two distinct lineages (hereafter named as North I and II) correspond to the two sections of *Ampelopsis *[58]. North I includes all species of section *Leeaceifoliae *with pinnately to bipinnately compound leaves. North II consists of taxa of section *Ampelopsis *with simple or palmately-divided or palmately-compound leaves. The Southern Hemisphere taxa (the African *Rhoicissus*, the South American *Cissus striata *complex, and the Australian *Clematicissus*) form a clade (the South group in Figure 2).

A total of 100 million generations (2 runs of 50 million generations each) are necessary to reach sufficient ESS. The *Ampelopsis *clade is estimated to have diverged from its close relatives in Vitaceae at 41.2 (23.4 - 61.0) Ma in the Eocene. The Bayesian estimates also suggest that all the other major clades of Vitaceae (e.g., *Vitis *- *Parthenocissus *- *Ampelocissus *clade, core *Cissus *clade, and *Cyphostemma *- *Cayratia *- *Tetrastigma*) had already diversified in the Eocene (Figure 2). Ages of major groups within the *Ampelopsis *clade obtained in our study are summarized in Table 1.

**Table 1:** Results of molecular dating and ancestral range reconstruction for major nodes within the *Ampelopsis *clade.

	-lnL	D	E	Node 0: Crown *Ampelopsis *clade	Node 1: Crown North I	Node 2: Stem South group	Node 3: Crown South group	Node 4: Crown *Cissus striata *complex - *Clematicissus*	Node 5: Disjunct
Molecular dating with BEAST (Ma)				41.2 (23.4-61.0)	21.8(6.2-26.3)	35.5(20.7-52.3)	30.5(16.9-45.9)	17.4(7.4-29.0)	19.2(6.7-22.3)
M0	32.42	0.003401	0.004318	N|NF (0.25)	A|N (0.81)	F|N (0.32)	S|F (0.22), U|F (0.21)	U|S (0.63)	N|A (0.47)
**M1**	**30.19**	**0.007085**	**0.003586**	**N|NS (0.61)**	**A|N (0.85)**	**S|N (0.92)**	**S|F (0.54)**	**U|S (0.79)**	**N|A (0.46)**, **N|E (0.36)**
M2	34.47	0.00986	0.008992	A|E (0.25), N|E (0.20)	A|N (0.51)	F|E (0.55)	F|F (0.39)	U|S (0.32)	N|E (0.51)
M3	32.94	0.007539	0.005598	A|AU (0.60)	A|N (0.68)	U|A (0.90)	U|F (0.42), U|U (0.41)	U|S (0.77)	N|A (0.72)
M4	30.65	0.006486	0.003784	N|NS (0.61)	A|N (0.84)	S|N (0.91)	S|F (0.51)	U|S (0.78)	N|A (0.46), N|E (0.35)
M5	30.89	0.006096	0.003555	N|NS (0.58)	A|N (0.84)	S|N (0.86)	S|F (0.51)	U|S (0.79)	N|A (0.48), N|E (0.35)
M6	33.3	0.007028	0.005918	A|AU (0.55)	A|N (0.66)	U|A (0.80)	U|U (0.40), U|F (0.37)	U|S (0.73)	N|A (0.65)

The nodes of interest are shown in Figure 2, and the likelihood scores (-lnL), and estimates of dispersal (D) and extinction (E) rates (events per million years) are given. M0 is a null model without constraints and M1-M6 are alternative models in the Lagrange analyses. Only the highest relative probability is shown. Bold text represents the model with a significantly better likelihood when compared with the other models tested (more than two log-likelihoods better)

**Figure 3:**
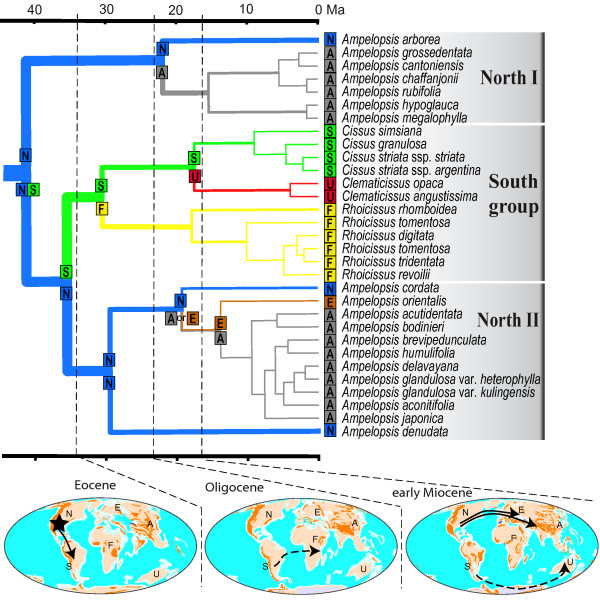
**Biogeographic scenario for the global disjunction of the *Ampelopsis *clade based on molecular dating and the best M1 model with the highest likelihood score in the ancestral range analyses**. Long distance dispersal is indicated as dash lines and migration as solid lines. The ancestral area of the *Ampelopsis *clade is shown with black stars on the maps. The tree branches and ranges on the tree are coded as follows: blue = North America (N); grey = eastern Asia (A); orange = Europe and central Asia (E); green = South America (S); yellow = Africa (F); and red = Australia (U).

Patterns of temporal and spatial distribution of the *Ampelopsis *clade are inferred using the maximum likelihood DEC method. We compare seven models (i.e., a null and six alternatives) for the six areas (Figure 1) and the effects of different models on likelihood reconstructions are shown in Table 1. Analyses based on M1, M4, and M5 typically have lower likelihood scores than other models and produced nearly identical results (Table 1). For example, all of them suggested that the ancestral range split at the stem South lineage is between North and South America (Node 2 in Table 1). Our results also suggest that the three models (M1, M4, and M5) are significantly different from the others (M2, M3, and M6) with scores over two log-likelihood units. The M1 model is suggested as the best one with the highest likelihood score, and this model is shown in Figure 3.

## Discussion

### North American origin

The *Ampelopsis *clade is composed of two distinct Laurasian lineages each disjunct between the Old and the New World and one South group with a Gondwana-like intercontinental disjunction: (Africa (Australia, and South America)) (Figure 3). The most likely model M1 in the DEC analyses suggested that the *Ampelopsis *clade had an early diversification in North America with a geographic split between N (North America) and NS (North and South America) (Table 1; Figure 3). The fact that most fossil records of the family including the oldest record in the Paleocene are found from North America [46] is consistent with this "out of North America" hypothesis. Although South America is inferred as part of the ancestral areas (Table 1), it seems less likely to be the ancestral area of the *Ampelopsis *clade than North America because there are very few fossils known before the Eocene of South America [47]. Furthermore, phylogenetic results also contradict the possibility of South American origin because the South American group is well embedded within the *Ampelopsis *clade (Figure 2). Asia also seems less likely than North America to be the ancestral area of the *Ampelopsis *clade, in spite of its highest extant species richness of the lineage. No seed records with ages younger than Oligocene are known from Asia [47].

The ancestral area for a taxon is usually expected to be correlated with high extant species richness. For example, eastern Asia usually has a higher level of species diversity and endemism, and has been suggested to be the ancestral area for many eastern Asian - eastern North American disjunct groups [59-63]. Donoghue and Smith (2004) found a predominance of directionality from Asia to the New World. Of the 29 lineages they analyzed with an eastern Asian and eastern North American disjunction, only one lineage showed directionality from eastern North America to eastern Asia. However, Wen *et al*. (2010) reported many more lineages with North American origins and subsequent migrations into eastern Asia, with 29 of the total 98 examined (30%) lineages migrated/dispersed from the New World to the Old World. It seems that eastern Asia has been over-emphasized as an ancestral area for Laurasian taxa due to its retention of the greater number of species [62,64]. North America is supported to have played an important role in the early evolution of the two *Ampelopsis *lineages in spite of the lower species diversity today in North America compared with eastern Asia. The lower species richness in North America is often explained by the hypothesis that both North America and eastern Asia were occupied by Boreotropical elements in the early Tertiary but North America suffered more severe extinctions with global cooling beginning in the late Eocene or Oligocene [10,63]. The high level of species diversity and endemism in Asia can also be attributed to secondary diversification due to habitat heterogeneity as well as a lower rate of extinctions in the late Tertiary [60,61].

### Diversification pattern in the Southern Hemisphere

The likelihood analyses using Lagrange based on M1, M4, and M5 suggest that the most likely route between the Northern and the Southern Hemisphere is from North to South America, although more options of connections are permitted in M4 (between Europe and Africa) and M5 (between eastern Asia and Australia) (Table 1 and Figure 3). *Ampelopsis *may have dispersed into South America via scattered continental and/or volcanic islands that connected North and South America at various times in the Tertiary [1,17], such as via the proto Greater Antilles (ca. 50 Ma) or via GAARlandia that existed around 33 - 35 Ma [17,65]. The separation of the southern lineage from its Laurasian ancestor at 35.5 (20.7 - 52.3) Ma in the late Eocene broadly coincides with a possible biological connection between North and South America around the Eocene-Oligocene boundary [66]. Fossil seeds found from the Eocene of South America are closely related to those of the Central American *Ampelocissus*, indicating the possible floristic connection between North and South America at that time [46].

Another possible migration route is that the Laurasian ancestors of the *Ampelopsis *clade reached Africa using the Boreotropical connection via the North Atlantic and Europe in the late Eocene to early Oligocene. However, this hypothesis is rejected by the Lagrange analysis (see M2 in Table 1). The model M4 with two possible connections between the Northern and Southern Hemisphere (N - S and E - F, Figure 1) did not support the European-African (E - F) route. Although the separation of the southern lineage from its Laurasian ancestor in the early Oligocene broadly coincides with the disruption of the Boreotropical flora around the Eocene-Oligocene boundary [66], both the shallow seas that separate Africa from Eurasia and the dry belt in northern Africa were barriers to biotic exchange between the two continents in the early to mid Tertiary [1,67,68]. The third hypothesis (the M3 model, Table 1) is that *Ampelopsis *entered the Southern Hemisphere via the Asian - Australian connection. This model seems quite unlikely based on our analyses (Table 1). The model M5 that permits two connections between Northern and Southern Hemisphere (N - S, A - U, Figure 1) also prefers the connection between North and South America (N - S) rather than the Asian - Australian connection (A - U). The availability of biotic interchange between Australia and Asia beginning at the Miocene [18] is too recent to support this scenario.

The divergence time 30.5 (95% HPD: 16.9 - 45.9) Ma in the early Oligocene was estimated for the first split between South America and Africa (node 3 in Figure 2 and Table 1). This time is well after the last possible connection of Africa and other southern landmasses at around 96-105 Ma [18,19]. We thus argue that long distance dispersal (LDD) is the most plausible mechanism for their southern intercontinental disjunction. Vitaceae taxa are usually dispersed by animals, especially birds [32,69-71]. All taxa in the *Ampelopsis *clade except the Australian *Clematicissus angustissima *bear fleshy berries that may have facilitated LDD. In particular, LDD has been recently accepted by a number of studies as the driving force for plant disjunctions in the Southern Hemisphere, especially for those with relatively recent divergence times [5,20,72-74]. Biogeographic studies on Vochysiaceae [75] suggested a LDD from South America to Africa in the Oligocene. Givnish et al. [76] showed that the single African genus *Maschocephalus *of Rapateaceae is of recent origin in the late Miocene and reached Africa from South America via LDD. Dispersals between Africa and South America have also been suggested for a number of well-studied taxa, such as in Melastomataceae [77], and Simaroubaceae [73].

Our results support that the southern lineage of *Ampelopsis *arrived in Australia from South America in the early Miocene (node 4 in Figure 2). Migration between Australia and South America may be alternatively explained by a trans-Antarctic exchange [12]. This Antarctic route existed during the late Cretaceous-early Tertiary and was interrupted only in the late Eocene (30-35 Ma) when the South Tasman Sea opened up between Australia and eastern Antarctica [19,78]. This route is supported by evidence from several plant groups, such as Annonaceae [79], and Sapotaceae [80]. Yet the split between the South American *Cissus striata *complex and the Australian *Clematicissus *in the early Miocene is too young to be explained by an Antarctic migration. LDD is the most plausible explanation for this disjunction. Finally, the disjunction of the two *Clematicissus *species in eastern and western Australia [30,81] may represent a relict distribution and their divergence time in the Pliocene is consistent with the aridification in central Australia at that time [82,83].

### Laurasian migrations

The DEC reconstruction suggests a North American - eastern Asian split (N|A) for the North I disjunction and a North American - eastern Asian or North American - Europe split (N|A or N|E) for the disjunction in the North II lineage (Table 1; Figure 3). The two Northern Hemisphere disjunctions may have involved the North Atlantic land bridges or the Bering land bridge from North America to eastern Asia [10,84]. We prefer to use the North Atlantic route because it is well supported in sect. *Ampelopsis *(North II in Figure 3). The southern North American *Ampelopsis denudata *diverged first, followed by the southeastern US *A. cordata*. The western Asian/southern European *A. orientalis *is then sister to the large Asian clade. The relative position of these areas in the cladogram is congruent with the migration of the lineage from North America to Europe across the North Atlantic land bridges, and the lineage then reached Asia subsequently (Figure 3).

The Bayesian molecular clock dating with fossil calibration suggests an early Miocene split of the two disjunct groups in *Ampelopsis *(nodes 1 and 5 in Figure 2). The divergence times are also consistent with the possibility of the North Atlantic migration route. Based on paleogeological, zoological, and botanical fossil evidence, Tiffney (1985b) argued for the importance of the North Atlantic land bridges to tropical or warm temperate species in the early Eocene to middle Miocene. A continuous belt of Boreotropical elements covered much of the southern part of North America, southern Eurasia, and northwestern Africa in the Eocene [9,66]. At that time, plant migrations through direct land connection or across limited water gaps were possible through the North Atlantic land bridges. Significant cooling during the Oligocene resulted in southward retreats and the extirpation of some lineages comprising this flora [64,85,86]. A gradual warming period occurred into the early Miocene, resulting in the expansion of some evergreen and thermophilic lineages in Europe and North America [87,88]. Dispersal of Boreotropical or warm temperate thermophilic elements, such as *Ampelopsis*, is therefore considered likely across the North Atlantic land bridges during this period. There are very few extant Vitaceae species in Europe, but many vitaceous seeds were reported in the early Tertiary of Europe [47,89]. Together, these lines of evidence strongly suggest that the *Ampelopsis *clade may have used the corridors via the North Atlantic land bridges as a pathway to reach Eurasia in the early Miocene.

## Conclusions

Our results suggest a complex history of diversification in the *Ampelopsis *clade to explain the global disjunctions that includes a North American origin, two Laurasian migrations, one migration into South America, and two post-Gondwanan LDDs. These findings may have general implications for the origin and diversification of plants with global disjunctions. Asia, Africa, or South America has often been suggested as the ancestral area for many intercontinental disjunct groups [4,90,91]. Evidence from the *Ampelopsis *clade suggests that North America may have played an important role in the origin of some modern flowering plants in spite of its often lower species diversity when compared with other areas, such as eastern Asia [92] or Africa [93]. Recent biogeographic analyses of several other groups appear to provide additional examples of North American origins (e.g., *Phryma *[94]; Simaroubaceae [73]). This study also highlights the importance of the North and South American route in the global diversification between the Northern and the Southern Hemisphere [95]. This route apparently played an important role in the wide distribution of many pantropical plants in the early Tertiary, such as Annonaceae [96,97], Malpighiaceae [91], and Rubiaceae [98].

Recent biogeographic analyses have underscored the relative importance of LDD to intercontinental disjunctions in the Southern Hemisphere than traditionally assumed [3,13]. The fit between area cladograms and the history of tectonic fragmentation might have been overstated [5]. If we accepted the results of our calibration analyses as absolute, rather than minimum ages, then the Southern Hemisphere clade is too young to have been achieved by the Gondwanan breakup. The Gondwana-like disjunction in the *Ampelopsis *clade was reconstructed to have a North American origin with an initial migration into South America and then dispersed from South America into Africa and Australia independently via LDD. A similar example from *Lycium *(85 spp., Solanaceae) was suggested a New World origin of *Lycium *with recent dispersal from the Americas to Africa, and then to eastern Asia [99-101]. Dispersal has been hypothesized to be the dominant pattern in this genus that has red, fleshy, bird-dispersed fruits [99]. On the other hand, however, the Laurasian lineages in *Ampelopsis *clade favor a vicariance migration pattern from North America via the North Atlantic land bridges to Eurasia. Our results thus support both the Laurasian migrations and the post-Gondwanan LDD to explain the global disjunction of the *Ampelopsis *clade.

## Authors' contributions

JW, ZLN, HS, and SRM conceived the ideas; JW, YM, QL and ZLN collected the materials; ZLN and YM analyzed the data; and ZLN and JW led the writing. All authors read and approved the final submission.

## Supplementary Material

Additional file 1 **Table S1. Voucher information and GenBank accession numbers of the Ampelopsis clade and representative taxa in Vitaceae**. Abbreviations of herbaria are as follows: KUN, Kunming Institute of Botany, Chinese Academy of Sciences; and US, the United States National Herbarium. Accession numbers beginning with JQ indicate sequences generated for this study and the others were obtained from GenBank. A dash means sequences missing. Click here for file
