# Supplementary material for: Evolution of the intercontinental disjunctions in six continents in the Ampelopsis clade of the grape family (Vitaceae)
Source: BMC Evol Biol. 2012 Feb 8;12:17. doi: 10.1186/1471-2148-12-17 (PMC3299610; doi:10.1186/1471-2148-12-17)
Supplement: Additional file 1 — Table S1. Voucher information and GenBank accession numbers of the Ampelopsis clade and representative taxa in Vitaceae. Abbreviations of herbaria are as follows: KUN, Kunming Institute of Botany, Chinese Academy of Sciences; and US, the United States National Herbarium. Accession numbers beginning with JQ indicate sequences generated for this study and the others were obtained from GenBank. A dash means sequences missing. [file 1471-2148-12-17-S1.DOC]

| Taxa | Voucher/Source | Locality | *trnL-F* | *atpB-rbcL* | *rps16* | *psbA-trnH* |
| --- | --- | --- | --- | --- | --- | --- |
| *Ampelocissus acapulcensis* (Kunth) Planch. | *Wen 8696* (US) | Mexico | JF437281 | JQ182472 | JQ182627 | JF437058 |
| *Ampelocissus africana* (Lour.) Merr. | *Luke & Luke 11449* (US) | Kenya, Makindu | JQ182553 | JQ182448 | JQ182603 | JQ182507 |
| *Ampelocissus africana* (Lour.) Merr. | *Luke & Luke 11536* (US) | Tanzania, Udzungwa Mountain | JQ182550 | JQ182444 | JQ182599 | JQ182504 |
| *Ampelocissus ascendiflora* Latiff | *Wen 8422* (US) | Malaysia, Selangor | AB234982 | JQ182430 | JQ182583 | — |
| *Ampelocissus elegans* Gagnep. | *Wen 7507* (US) | Singapore, Bukit Timah Nature Reserve | AB234981 | JQ182427 | JQ182581 | — |
| *Ampelocissus elephantina* Planch. | *Wen 9583* (US) | Madagascar | — | HM585516 | HM585792 | HM585659 |
| *Ampelocissus erdwendbergii* Planch. | *Wen 8697* (US) | Mexico | JQ182569 | JQ182471 | JQ182626 | JQ182527 |
| *Ampelocissus javalensis* (Seem.) W.D. Stevens & A. Pool | *Wen 6920* (US) | Costa Rica | AB234984 | AB234911 | AB234943 | — |
| *Ampelocissus obtusata* (Welw. ex Baker) Planch. | *Luke & Luke 11590* (US) | Tanzania, Inyonga | JQ182556 | JQ182457 | JQ182612 | JQ182510 |
| *Ampelocissus thyrsiflora* (Blume) Planch. | *Deden 870* (US) | Indonesia, SE Sulawesi | JQ182546 | JQ182438 | JQ182593 | JQ182499 |
| *Ampelopsis aconitifolia* Bunge | *Wen 8518* (US) | China, Beijing | JQ182560 | JQ182461 | JQ182616 | JQ182517 |
| *Ampelopsis acutidentata* W.T. Wang | *Wen et al. (Tibet-MacArthur) 3479* (KUN, US) | China, Yunnan | JQ182561 | JQ182462 | JQ182617 | JQ182518 |
| *Ampelopsis arborea* Kochne | *Wen 7164* (US) | USA, Alabama | AB234990 | — | AB234946 | JQ182487 |
| *Ampelopsis bodinieri* (H. Lév. & Vaniot) Rehder | *Wen 9003* (US) | China, Shaanxi | JQ182562 | JQ182463 | JQ182618 | JQ182519 |
| *Ampelopsis cantoniensis* K. Koch | *Wen 10810* (US) | Vietnam | JQ182545 | JQ182437 | JQ182592 | JQ182498 |
| *Ampelopsis chaffanjonii* (H. Lev.) Rehder | *Wen 9382* (US) | China, Guangxi | JQ182570 | JQ182475 | JQ182630 | JQ182528 |
| *Ampelopsis cordata* Michx. | *Wen 9700* (US) | USA, Texas | JQ182558 | JQ182459 | JQ182614 | JQ182513 |
| *Ampelopsis delavayana* Planch. ex Franch. | *Wen 9377* (US) | China, Guangxi | JF437287 | JQ182476 | JQ182631 | JF437065 |
| *Ampelopsis denudata* Planch. | *Wen 8695* (US) | Mexico | JQ182577 | JQ182483 | JQ182638 | JQ182534 |
| *Ampelopsis glandulosa* var. *hancei* (Planch.) Momiy. | *Wen 8289* (US) | Philippines | JQ182571 | JQ182477 | JQ182632 | JQ182529 |
| *Ampelopsis glandulosa* var. *heterophylla* (Thunb.) Mom. | *Wen 9380* (US) | China, Guangxi | JF437289 | JQ182474 | JQ182629 | JF437067 |
| *Ampelopsis glandulosa* var. *kulingensis* (Rehd.) Mom. | *Wen 9361* (US) | China, Hunan | JQ182572 | JQ182478 | JQ182633 | JQ182530 |
| *Ampelopsis grossedentata* (Hand.-Mazz.) W.T. Wang | *Wen 9336* (US) | China, Hunan | JQ182573 | JQ182479 | JQ182634 | JQ182531 |
| *Ampelopsis humulifolia* Bge. | *Wen 8519* (US) | China, Beijing | JQ182563 | JQ182464 | JQ182619 | JQ182520 |
| *Ampelopsis hypoglauca* (Hance) C.L. Li | *Wen 8195* (US) | China, Chongqing | AB235000 | JQ182431 | JQ182584 | JQ182490 |
| *Ampelopsis japonica* (Thunb.) Makino | *Wen s.n.* (US) | Cult. in Washington D.C. | JQ182538 | — | JQ182585 | JQ182491 |
| *Ampelopsis megalophylla* Diels & Gilg | *Wen 9035* (US) | China, Shaanxi | JQ182564 | JQ182465 | JQ182620 | JQ182521 |
| *Ampelopsis orientalis* Planch. | *Al-Shehbaz 0687* (US) | Turkey | JQ182565 | JQ182466 | JQ182621 | JQ182522 |
| *Ampelopsis rubifolia* (Wall.) Planch. | *Wen 9285* (US) | China, Hunan | JF437293 | JQ182473 | JQ182628 | JF437072 |
| *Cayratia cordifolia* C.Y. Wu | *Wen 10548* (US) | China, Yunnan | HM585934 | HM585518 | HM585794 | HM585661 |
| *Cayratia geniculata* (Blume) Gagnep. | *Wen 10680* (US) | Indonesia, West Java | JQ182575 | JQ182481 | JQ182636 | JQ182532 |
| *Cayratia japonica* (Thunb.) Gagnep | *Shui et al. 81847* (KUN, US) | China, SE Yunnan | JQ182578 | JQ182484 | JQ182639 | JQ182535 |
| *Cayratia maritima* Jackes | *Wen 10701* (US) | Indonesia, Papua | JQ182576 | JQ182482 | JQ182637 | JQ182533 |
| *Cayratia mollissima* (Wall.) Gagnep. | *Wen 8403* (US) | Malaysia, Pahang | HM585938 | HM585522 | HM585798 | HM585665 |
| *Cayratia* sp. | *Wen 10301* (US) | Indonesia, SE Sulawesi | JQ182547 | JQ182439 | JQ182594 | JQ182500 |
| *Cayratia wrayi* (King) Gagnep. | *Wen 10913* (US) | Vietnam | JQ182544 | JQ182436 | JQ182591 | JQ182497 |
| *Cissus adnata* Roxb. | *Wen 10519* (US) | China, Yunnan | JQ182579 | JQ182485 | JQ182640 | JQ182536 |
| *Cissus albiporcata* Masinde & L. E. Newton | *Luke & Luke 11456* (US) | Kenya, Chyulu Plains | JF437304 | JQ182442 | JQ182597 | JF437087 |
| *Cissus aralioides* Planch. | *Aplin s.n.* (US) | Cult. in Belgium National Bot Garden | JQ182554 | JQ182455 | JQ182610 | JQ182508 |
| *Cissus erosa* Rich. | *Wen 8586* (US) | Peru, Pasco | HM585942 | HM585526 | HM585802 | HM585668 |
| *Cissus granulosa* Ruiz & Pav. | *Wen 8611* (US) | Peru, Pasco | JQ182543 | JQ182435 | JQ182590 | JQ182496 |
| *Cissus integrifolia* (Baker) Planch. | *Luke & Luke 11475* (US) | Kenya, Shimba Hills | JQ182551 | JQ182445 | JQ182600 | JQ182505 |
| *Cissus javana* DC. | *Shui et al. 81970* (KUN, US) | China, SE Yunnan | JQ182580 | JQ182486 | JQ182641 | JQ182537 |
| *Cissus nodosa* Blume | *Wen 10713* (US) | Indonesia, Papua | HM585945 | HM585529 | HM585805 | HM585671 |
| *Cissus phymatocarpa* Masinde & L.E. Newton | *Luke & Luke 11474* (US) | Kenya, Diani Forest | JF437311 | JQ182452 | JQ182607 | JF437095 |
| *Cissus pileata* Desc. | *Wen 9662* (US) | Madagascar | JQ182557 | JQ182458 | JQ182613 | JQ182512 |
| *Cissus producta* Afzel. | *Luke & Luke 11528* (US) | Tanzania, Udzungwa Mountain | JF437312 | JQ182447 | JQ182602 | JF437096 |
| *Cissus repens* Lam. | *Shui et al. 81807* (KUN, US) | China, Yunnan | HM585946 | HM585530 | HM585806 | HM585672 |
| *Cissus simsiana* Schult. & Schult. f. | *Nee & Wen 53805* (US) | Bolivia, Santa Cruz | JQ182539 | — | JQ182586 | JQ182492 |
| *Cissus striata* ssp. *argentina* (Suess.) Lombardi | *Nee & Wen 53854* (US) | Bolivia, Santa Cruz | JQ182540 | JQ182432 | JQ182587 | JQ182493 |
| *Cissus striata* ssp. *striata* Ruiz & Pav. | *Wen 7355* (US) | Chile, Concepcion | AB235018 | JQ182428 | JQ182582 | JF437104 |
| *Cissus trianae* Planch. | *Nee & Wen 53942* (US) | Bolivia, Santa Cruz | JQ182541 | JQ182433 | JQ182588 | JQ182494 |
| *Cissus verticillata* (L.) Nicolson & C.E. Jarvis | *Wen 10165* (US) | Indonesia | JQ182542 | JQ182434 | JQ182589 | JQ182495 |
| *Clematicissus angustissima* (F. Muell.) Planch. | *Rossetto et al., 2002* | Australia, Western Australia | JQ182574 | JQ182480 | JQ182635 | — |
| *Clematicissus opaca* (F. Muell.) Jackes & Rossetto | *Rossetto et al., 2002* | Australia, Queensland | JQ182548 | JQ182440 | JQ182595 | JQ182501 |
| *Cyphostemma kilimandscharicum* (Gilg) Wild & R.B. Drumm. | *Luke & Luke 11469* (US) | Kenya, Chyulu Hills | JF437327 | JQ182451 | JQ182606 | JF437112 |
| *Cyphostemma maranguense* (Gilg) Desc. | *Luke & Luke 11468* (US) | Kenya, Chyulu Hills | JF437329 | JQ182449 | JQ182604 | JF437114 |
| *Cyphostemma serpens* (Hochst. ex A. Rich.) Desc. | *Luke & Luke 11447* (US) | Kenya, Kiboko | JQ182552 | JQ182446 | JQ182601 | JQ182506 |
| *Cyphostemma thomasii* (Gilg & Brandt) Descoings | *Luke & Luke 11448* (US) | Kenya, Makindu | JF437331 | JQ182450 | JQ182605 | JF437117 |
| *Leea gonioptera* Lauterb. | *Wen 10711* (US) | Indonesia, Papua | JQ182559 | JQ182460 | JQ182615 | — |
| *Leea guineensis* G. Don | *Wen 80_84* (US) | Cult. in Hawaii, originally from Africa | JQ182549 | JQ182441 | JQ182596 | JQ182503 |
| *Leea indica* (Burm. f.) Merr. | *Wen 10910* (US) | Vietnam | HM585953 | HM585537 | HM585813 | HM585679 |
| *Parthenocissus chinensis* C.L. Li | *Nie & Meng 470* (KUN, US) | China, Sichuan | HM223270 | HM223381 | HM223327 | JQ182502 |
| *Parthenocissus feddei* (Lévl.) C.L. Li | *Zhang 319* (US) | China, Hunnan | HM223307 | HM223416 | HM223359 | JQ182526 |
| *Parthenocissus quinquefolia* (L.) Planch. | *Wen 9711* (US) | USA, Texas | HM223258 | HM223368 | HM223315 | JQ182514 |
| *Parthenocissus semicordata* (Wall.) Planch. | *Wen et al. (Tibet-MacArthur) 887* (KUN, US) | China, Xizang | HM223271 | HM223382 | HM223328 | JQ182511 |
| *Parthenocissus suberosa* Hand.-Mazz. | *Nie & Meng 358* (KUN, US) | China, Guizhou | HM223273 | HM223384 | HM223330 | JF437134 |
| *Parthenocissus vitacea* (Knerr) Hitchc. | *Wen 10488* (US) | Canada, Quebéc | HM223295 | HM223407 | HM223352 | HM585681 |
| *Rhoicissus digitata* Gilg & Brandt | *Gerrath s.n.* (US) | cult. in Iowa, USA | AB235047 | JQ182429 | AB234966 | JQ182489 |
| *Rhoicissus revoilii* Planch. | *Luke & Luke 11698* (US) | Kenya, Magadi | JQ182555 | JQ182456 | JQ182611 | JQ182509 |
| *Rhoicissus rhomboidea* Planch. | *Wen 6673* (US) | Cult. in Missouri Bot. Gard., USA | AB235048 | AB234931 | AB234967 | JQ182488 |
| *Rhoicissus tomentosa* (Lam.) Wild & R.B. Drumm. | *Aplin 19656252* (US) | Cult. in Belgium National Bot Garden | JF437342 | JQ182454 | JQ182609 | JF437139 |
| *Rhoicissus tomentosa* (Lam.) Wild & R.B. Drumm. | *Wen 10076* (US) | South Africa, KwaZulu Natal | HM223252 | HM223362 | HM223309 | JQ182516 |
| *Rhoicissus tridentata* (L. f.) Wild & R.B. Drumm. | *Luke & Luke 11453* (US) | Kenya, Chyulu Hills | JF437341 | JQ182443 | JQ182598 | JF437138 |
| *Tetrastigma caudatum* Merr. & Chun | *Wen 10812* (US) | Vietnam, Vinh Phuc | HM585967 | HM585551 | HM585827 | HM585691 |
| *Tetrastigma ceratopetalum* C.Y. Wu | *Shui et al. 81836* (KUN, US) | China, SE Yunnan | HM585937 | HM585521 | HM585797 | HM585664 |
| *Tetrastigma erubescens* Planch. | *Wen 10968* (US) | Vietnam, Guangnam | HM585987 | HM585571 | HM585847 | HM585711 |
| *Tetrastigma henryi* Gagnep. | *Wen 10518* (US) | China, Yunnan | HM586002 | HM585586 | HM585862 | HM585724 |
| *Tetrastigma obovatum* (M.A. Lawson) Gagnep. | *Wen 10567* (US) | China, Yunnan | HM586024 | HM585608 | HM585883 | HM585746 |
| *Tetrastigma serrulatum* (Roxb.) Planch. | *Wen 10532* (US) | China, Yunnan | HM586003 | HM585587 | HM585863 | HM585725 |
| *Tetrastigma sichouense* C.L. Li | *Wen 10547* (US) | China, Yunnan | HM586046 | HM585631 | HM585905 | HM585768 |
| *Vitis aestivalis* Michx. | *Wen 10428* (US) | USA, Delaware | HM223286 | HM223398 | HM223343 | JQ182515 |
| *Vitis davidiana* (Carrière) G. Nicholson | *Wen 9060* (US) | China, Shaanxi | JQ182568 | JQ182470 | JQ182625 | JQ182525 |
| *Vitis flexuosa* Thunb. | *Wen 8540* (US) | Japan, Chiba - Ken | JQ182567 | JQ182469 | JQ182624 | JQ182524 |
| *Vitis mengziensis* C.L. Li | *Nie & Meng 415* (KUN, US) | China, Yunnan | HM223276 | HM223387 | HM223333 | JF437158 |
| *Vitis popenoei* Fennell | *Wen 8724* (US) | Mexico | HM586072 | HM585657 | HM585930 | HM585790 |
| *Vitis riparia* Michx. | *Wen 8658* (US) | USA, Virginia | JF437357 | JQ182453 | JQ182608 | JF437165 |
| *Vitis rotundifolia* Michx. | *Wen 11087* (US) | USA, Arkansas | JF437358 | JQ182468 | JQ182623 | JF437166 |
| *Vitis vulpina* L. | *Wen 11082* (US) | USA, Arkansas | JQ182566 | JQ182467 | JQ182622 | JQ182523 |
| *Yua thomsonii* (M.A. Lawson) C.L. Li | *Nie & Meng 469* (KUN, US) | China, Sichuan | HM223277 | HM223389 | HM223335 | JF437171 |
